# Work and breast milk feeding: a qualitative exploration of the experience of lactating mothers working in ready made garments factories in urban Bangladesh

**DOI:** 10.1186/s13006-020-00338-0

**Published:** 2020-11-07

**Authors:** A M Rumayan Hasan, George Smith, Mohammad Abdus Selim, Shahinoor Akter, Nazib Uz Zaman Khan, Tamanna Sharmin, Sabrina Rasheed

**Affiliations:** 1 Health Systems and Population Studies Division (HSPSD), icddr,b, Universal Health Coverage, 68 Shaheed Tajuddin Ahmed Sarani, Mohakhali, Dhaka, 1212 Bangladesh; 2 Fragments Magazine, Dhaka, Bangladesh; 3 Faculty of Health and Medicine, Schools of Medicine and Public Health, The University of Newcastle, Newcastle, Australia; 4 Department of Anthropology, Jagannath University, Dhaka, Bangladesh; 5 Schools of Health and Life Sciences, Federation University, Ballarat, Victoria 3350 Australia; 6 Monitoring Evaluation Research and Learning (MERL), Plan International, Dhaka, Bangladesh

**Keywords:** Breastfeeding, Working women, Bangladesh, Barriers, RMG sector, Formative research, Expressed breast milk

## Abstract

**Background:**

In Bangladesh 65% of children under 6 months of age were exclusively breastfed with maternal employment being a risk factor that has jeopardized exclusive breastfeeding. As Ready Made Garment (RMG) factories have been the largest employer of low income women in Bangladesh, the objective of our study was to explore the barriers and facilitators of breastfeeding and perceptions about use of expressed breast milk among mothers who worked in the RMG sector.

**Methods:**

This formative research was conducted during July–September 2015 in two slums of Dhaka among RMG workers who were mothers and the caregivers of 0–12 month old infants. Qualitative data was obtained from purposively selected participants of 8 in-depth interviews and 4 focus group discussions (mothers and caregivers), and 2 key informant (RMG factory official) interviews. Mothers were from multiple RMG factories while factory officials were from a single factory. Thematic analysis was conducted.

**Results:**

The main themes of qualitative exploration were knowledge and experience of breastfeeding; structural barriers (home and workplace); consequences of inadequate breastfeeding; and perception and experience of using expressed breast milk. Despite knowledge both of the benefits of breast milk and of the importance of breastfeeding for 6 months, most mothers introduced formula as early as 2 months to prepare for their return to work. Barriers such as excessive workload, inadequate crèche facilities at work, and lack of adequate caregivers at home impeded exclusive breastfeeding. Mothers and caregivers had very little knowledge about the use of expressed breast milk and were concerned about contamination.

**Conclusion:**

As RMG factories are the largest employer of low-income women in Bangladesh, facilitating RMG factory working mothers’ ability to use breast milk could help to promote infant health and help women remain in the workforce.

## Background

Despite the benefits of breastfeeding, globally only 41% of children under age 6 months are exclusively breastfed [1]; in Bangladesh the rate is 65% [2]. In many studies maternal employment and social, economic and structural barriers have been shown to reduce the duration of exclusive breastfeeding [3–6]. The impact of structural barriers is more pronounced for those in poverty and lacking in education [7]. Structural barriers include maternal employment, maternal workload, work environment, work policies, social support, and negative societal perception about breastfeeding in public. In low income countries, limited social support, mother’s time constraints, household food insecurity and poverty often makes breastfeeding difficult [8, 9]. Structural barriers also extend into the health systems. Health policies and attitudes and practices of healthcare providers have been shown to affect breastfeeding practices [10–14]. In Bangladesh there has been a steady rise in female employment (among all people employed) in industries from 8.6% in 2000 to 16.8% in 2017 [15]. The Ready Made Garment (RMG) industry has been Bangladesh’s largest employer of women, employing an estimated 4.2 million women from low income households. Fifty-five to 60% of women who have been employed in RMG factories were of reproductive age [16]. Working mothers have had less opportunity to breastfeed on demand especially if workplaces do not provide crèches as this forces mothers to leave their infants at home. Long work hours and lack of crèches has led to early introduction of formula, reflected by low exclusive breastfeeding rates (10%) among RMG factory workers [17]. To help create a supportive environment, the Bangladesh Labour Act [18] mandates 4 months of paid maternity leave for women working in formal industries. The Labour Act also requires companies employing at least 40 workers to provide childcare facilities for children up to 6 years of age; however industry compliance has been inconsistent [17]. Despite legal rights, researchers have reported that female garment workers face violence in the workplace, difficult job conditions (including long working hours), stress caused by excessive workload and anxiety [19–21]. Researchers have also reported that RMG workers have limited access to government hospitals, that only large factories have medical services and that these services have been deemed inadequate to meet their needs [21, 22].

In high income countries, the wide availability of breast pumps has provided working mothers with a greater ability to express their breast milk while at work [23]. Working mothers given the opportunity to pump their breast milk, are more likely to breastfeed to 6 months of age and beyond [24]. However, even in high income countries unsupportive work environments can still lead to early weaning [25, 26]. To design interventions to promote the use of expressed breast milk, it is necessary to understand the breastfeeding experiences of mothers and to identify culturally appropriate and feasible ways of implementing interventions in the local context. In our study, therefore, we explored the facilitators and barriers of breastfeeding and perceptions about using expressed breast milk among mothers working in the RMG sector. Insights from our study will contribute to the design of programs to promote breastfeeding among low income working mothers in Bangladesh and similar contexts.

## Methods

### Study design

This was formative research conducted to explore the barriers and facilitators of breastfeeding and to identify culturally appropriate and feasible ways to promote the use of expressed breast milk by mothers working in RMG factories. The study population was mothers working in RMG factories with infants below 12 months of age as well as other caregivers of the infants.

### Study site

The study was conducted from July–September 2015 in the *Savar* and *Saat-tola* slum areas of Dhaka city where many female RMG workers resided*.*

### Data collection

Data were collected using qualitative methods of in-depth Interviews (IDIs), key informant interviews (KIIs), and focus group discussions (FGDs). The IDIs were conducted to understand infant and young child feeding practices and cultural perspectives about using expressed breast milk among mothers of infants and young children. FGDs were used to verify the feeding practices of working women and to explore community norms around using expressed breast milk. KIIs were used to understand the structural barriers to breastfeeding for mothers working in RMG factories from the factory management perspective (Table 1).

**Table 1:** Methods used and sample size

Method	Respondent type	Number of participants
IDIs	Ex-RMG workers, current RMG workers and RMG workers currently on maternity leave	8
FGDs	Current RMG workers, RMG workers currently on maternity leave and caregivers	25 (4 FGDs)
KIIs	Welfare officer and line supervisor of RMG factory	2

*IDI* In-Depth Interviews, *FGD* Focus Group Discussion, *KII* Key Informant Interview

### Sample and participants

Maximum variation sampling [27] was used to select mothers for in depth interviews (IDIs) and focus group discussions (FGDs). Participants were mothers of infants < 1 year of age who were working in RMG factories or on maternity leave from RMG factories or had recently quit RMG factory jobs. Initially we purposively selected 2 RMG factories next to slums where there were cooperative managers willing to assist us in recruiting IDI participants based on our criteria. After we conducted a few IDIs, our interviewees helped us identify additional participants for both IDIs and FGDs, who were their neighbors and/or relatives. Purposive sampling was used to select other caregivers for FGDs. These other caregivers were grandmothers, sisters or female relatives who cared for the infants. There was no overlap between participants of FGDs and IDIs which helped us cover diverse perspectives and experiences. KIIs were conducted in one factory willing to partner with us to understand the structural barriers from the factory management perspective.

We held FGDs on weekends and holidays when participants were more likely to be available at their residences. KIIs were conducted at RMG factories. Issues that had not been considered initially but emerged during data collection and preliminary data analysis were incorporated into the subsequent guidelines, resulting in our adding a few additional questions to the guidelines for both KIIs and FGDs.

The field team consisted of 3 female researchers with master degrees in anthropology and social science and 5–6 years’ experience collecting qualitative data. They were trained and supervised by an anthropologist (RH) and a public health nutritionist (SR).

### Ethical consideration

The study received ethical approval from the Ethical Review Committee of the **International Centre for Diarrhoeal Disease Research, Bangladesh** (icddr,b) in September 2015. All study participants provided informed verbal and written consent. We did not collect any personal identification data from participants; instead we assigned a code to each participant based on their group and area. Audio recordings and transcript data were kept in separate password-protected files.

### Data analysis

**Fig. 1:**
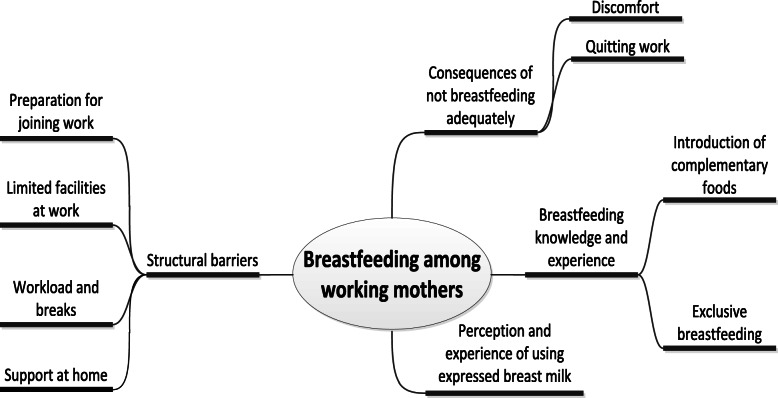
Themes of the qualitative exploration

All interviews and discussions were audio recorded and then transcribed verbatim in Bengali by the researchers. The data were analyzed thematically [28]. The analytic process entailed gaining an understanding of the data through repeated readings of the transcripts. Three investigators (RH, SA, AS) coded the transcripts independently. They met regularly to discuss and resolve any discrepancies in the coding. Themes were identified, amended, refined and named (Fig. 1).

Triangulation – the use of at least two methods of data collection to verify findings with the goal of ensuring that a biased person or group is not driving the findings – was performed using the different sources of both data and methods. The research team held regular peer debriefing sessions to help understand the issues and interpret the findings. Nvivo10 software was used to sort, manage and code the data [29].

## Results

### Characteristics of the participants

Twenty-seven mothers working at RMG factories participated in the study, with more than half 21–25 years of age. The majority of mothers had infants less than 6 months old and had female relatives who were caregivers for their children. For the FGD made up of caregivers, we purposefully selected six women (Table 2). Our study had two people who were key informants. They both worked at the same RMG factory, one as a line supervisor and the other as the factory’s sole welfare officer.

**Table 2:** Characteristics of the participants

Characteristics	IDI with mothers (***n*** = 8)	FGD with mothers (***n*** = 19)	FGD with caregivers (***n*** = 6)
***Age group***			
21–25	5	9	0
26+	3	10	6
***Years of schooling***			
0–5	5	10	5
6+	3	9	1
***Husband’s profession***			
Service	4	12	–
Day Labor	4	7	–
***Mother’s job experience (years)***			
1–5	3	7	–
6–10	4	7	–
11+	1	5	–
***Household income (****USD****)***			
100–150	3	7	–
151–200	4	5	–
200+	1	7	–
***Child’s age***			
≤ 6 months	5	15	–
6+ months	3	4	–
***Alternative caregiver (Relation to child)***			
Grandmother	0	0	4
Other relatives	0	0	2

### Breastfeeding knowledge and experience

#### Exclusive breastfeeding

All participants talked about breast milk being rich and nutritious and thought it was very important for infant health and development. They also mentioned the safety of breast milk:

*Did you not hear that there is no substitute (for breast milk) … It is true. Breast milk has no bhejal (contaminant).* [IDI 4_currently working mother]

All participants knew that one should feed breast milk only for at least the first 6 months. As one mother said:

*Feeding breast milk [up to six months] is good. Mother’s milk is nutritious. Other foods are not the same as breast milk. We are aware now* . . *. We learnt from TV.* [FGD 2_mother on maternity leave]

All the participants mentioned learning about breastfeeding for 6 months from their educated and experienced relatives and neighbors and from health professionals. Despite widespread knowledge of the need for breastfeeding during the first 6 months of life, when mothers were asked if other foods could be given during this time, some talked about the need to give infants water and sugar water to quench thirst. Though, many mothers thought that, ideally, complementary foods should be introduced only after six months.

#### Early introduction of complementary foods

Despite knowing that breast milk was the ideal food and that it was important to exclusively breastfeed for 6 months, most mothers working in RMG factories introduced formulas such as *Lactogen-1* or *Biomil* as early as 2 months after giving birth. As one mother who talked about the link between infant feeding choices and work said:

*The rule is to feed (breast milk) for six months but I can’ t*. . *. I have to work so I feed (formula).* [FGD 1_currently working mother]

Breast milk inadequacy led to early introduction of complementary foods early in a few cases:

*Breast milk is the best but when (the) baby does not get it she cries (with hunger) and then other foods are needed.* [FGD 3_currently working mother]

A few mothers knew that introducing complementary foods increased the risk of diarrhoea for infants and complained that their infants were often sick. They also expressed concern about the safety of the ingredients in formula:

*Read the ingredients (on formula container). In 100g of formula* . . *. one fourth (of the weight of the formula) is medicine. In a 400g container there is four times more medicine* . . *. Isn’t it too much? How can an infant tolerate (this amount of medicine)?* [IDI 4_currently working mother]

Most mothers who were concerned about introducing formula also talked about the risk associated with the accessories used to feed formula. They were aware that unclean bottles could cause disease:

*Bottles have germs. After a feed if the bottle is left for even two minutes, germs accumulate* . . *. Later if the bottle is used for another feed (without washing)* . . *. she (the infant) can become sick.* [FGD 1_currently working mother]

All mothers spoke about the inconvenience of making complementary foods and maintaining the accessories given that they resided in rented rooms with communal kitchens shared with 20–30 people. Often they had to wait for their turn to cook while their babies cried. As one caregiver explained:

*The baby starts crying if we are late (in feeding); this is natural . . . We sometimes can’t tolerate (the crying) . . . What can I do, we have to share the kitchen . . . I have to wait (my turn) . . . The baby suffers.* [FGD 4_caregiver]

For most mothers the cost of formula was a major problem. Mothers perceived that the price of formula had increased over time and that a container had risen in cost to US $5–6. As the child grew, the number of containers of formula mothers had to buy every month increased, which put significant pressure on family resources:

*This is an extra cost . . . It gets costlier everyday . . . Before I had money left over (from my salary) after expenses. Now I have no money left. Sometimes I have to borrow.* [FGD 3_currently working mother]

### Structural barriers impeding breastfeeding

#### Preparation for joining the workforce

Many mothers talked about initiating formula as early as 2 months after birth to prepare for their return to work after 3–4 months of maternity leave. Few RMG factories had creche facilities that allowed mothers to bring their infants to work, so mothers were forced to leave their infants at home with a caregiver. To prepare for the long work day separation, mothers started formula feeding early to accustom their infants. To choose which formula to buy they consulted their relatives, neighbors, co-workers and doctors. One mother said:

*I have started feeding my child Lactogen-1. I feed a little (of the formula) every day. When I join work I have to start feeding (formula) more often as I will be in the (factory) the whole day. I will breastfeed when I come home in the evening.* [FGD 1_currently working mother]

#### Limited facilities at work

A few of the mothers spoke about lack of both privacy and appropriate spaces as barriers to breastfeeding at work. Mothers said that supervisors allowed breaks for breastfeeding once or twice a day for 15–20 min when the infant could be brought to them. However, mothers could not actually breastfeed as no private space was allotted in the factory:

*They (supervisors) would allow me to breastfeed. But I cannot do it (at work). There is no place covered by (a) curtain in our factory where I can (breastfeed). If I want to breastfeed I have to do it at the gate (of the factory). Is it possible?* . . *. (There is) no privacy.* [FGD 3_currently working mother]

In factories where there was space and privacy for breastfeeding a few mothers who lived nearby made arrangements for caregivers to bring their infants during breaks. As one working mother said:

*There are some who have caregivers (at home)* . . *. They bring the baby to (the) office (for breastfeeding). I tell my line supervisor, “My child is here; let me go downstairs (to breastfeed).” He says, “No problem. It’s your work you have to finish.” They don’t have any problem. Mothers can breastfeed at the crèche or the doctor’s room.* [IDI 4_currently working mother]

Despite the availability of breastfeeding breaks, a few mothers had doubts about being able to avail these breaks regularly:

*If I tell my boss that I need to go (to breastfeed) then he will allow me to go for one, two or three days. Will he allow this everyday? No, this will hamper their work.* [IDI 1_currently working mother]

The factory management, however, disagreed and claimed that they had rules allowing breastfeeding breaks twice a day for infants who were brought to the factory during a mother’s break time.

Only one respondent mentioned that she had heard about factories that had crèche facilities where mothers could keep their children and breastfeed:

*Some people keep their babies in the office* . . *. they have the facility. Mothers come every 2-3 hours and breastfeed. Two caregivers look after the babies (in the crèche).* [IDI 4_currently working mother]

#### Workload and inadequate breaks

For all study participants working in RMG factories, the nature and pressure of the work were barriers to taking scheduled breaks. Although RMG workers were supposed to work 8 h days, when there were production deadlines they often worked much longer hours and struggled to take scheduled breaks. The tight schedules impeded daytime breastfeeding. According to one mother:

*I don’t get any break (at work). No one can replace me* . . *. I have to continuously run the machine. There is a break (at lunch) when I can breastfeed but sometimes I can go home and sometimes I can’t. I often reach home late after doing overtime so the whole day I can’t breastfeed.* [FGD 3_currently working mother]

As many of the RMG factory tasks were performed in an assembly line, if 1 person took a break the entire line had to stop. Some factories seemed to have a mechanism to replace mothers during their breaks; others did not. When mothers’ lactation breaks affected production, it put their jobs in jeopardy. One RMG worker explained:

*When there is (a) heavy workload if I go (for a break), you and others in the line have to wait (for me to come back). We work in a schedule and there is no scope (for) wasting time. They don’t allow me 10 minutes to breastfeed. I am in trouble (at work if I try to breastfeed).* [FGD 3_currently working mother]

Participants who managed to go home at lunch time found that an hour’s break was inadequate for them to travel home, eat lunch, breastfeed and return to work on time. And the longer the commute, the more difficult the schedule became.

#### Support at home

Since many factories had inadequate facilities for infants, mothers relied on female family members such as sisters, sisters-in-law, mothers or mothers-in law or older women in the neighborhood they paid to look after their children. To be able to breastfeed during the day mothers needed a reliable caregiver who could bring the baby to the factory at designated times. All the mothers who joined work had a caregiver but not all these caregivers were able to bring the infants to the factories. As one mothers said:

*My neighbor next door (an elderly woman) looks after my child. I wish someone could bring my baby to work. I could have breastfed her at work. There is no one who can bring her.* [IDI 4_currently working mother]

### Consequences of not being able to breastfeed adequately

#### Discomfort

When lactating mothers went a whole day without breastfeeding, they reported physical and mental discomfort which diminished their work productivity. A few mothers reported pain from breast engorgement:

*I face difficulty (at work). Mostly my breast feels heavy, they ache a lot* . . *. Milk comes out and wet(s) my clothes. I have to go the toilet to press and throw out (breast milk). Work piles up (because of the breaks) and I am under pressure.* [IDI 1_currently working mother]

Mothers explained the effects of breast engorgement on the quality of breast milk and on their own health:

*I know that breast milk becomes poisonous and curdles (if not fed frequently). Elders say that (milk) can cause stomach problems (for the infant). (A) mother’s breast hurts, becomes hard, engorged. She has high fever* . . *. many things happen. Sometimes she has to take time off.* [FGD 1_currently working mother]

Beyond the physical discomfort a few mothers also felt guilty when work prevented them from breastfeeding. A grandmother talked about her daughter’s situation:

*It is not my preference (to feed formula)* . . *. My daughter’s milk flows out* . . *. She needs to throw it out 3-4 times. She cries everyday (in despair). It is our fate that we can’t feed (breast milk) to our baby.* [FG 4_caregiver]

#### Quitting work

Most mothers of infants who quit their RMG factory jobs did so because of inhospitable work environments and a lack of quality caregivers at home. These mothers struggled to balance the need to ensure optimal feeding and care for their children with the demands of their jobs. This struggle was clear from the statements of RMG workers who quit their jobs after maternity leave:

*Only a mother knows when a child is hungry … Grandmothers don’t understand … That’s why I left (the job).* [IDI 2_ex-working mother]

*There is no place (a crèche) for kids at work. I could not keep him (with me). He is only two months old, he needs breast milk. If I work I will have to keep him at home and feed him formula* . . *. During the day I won’t be able to breastfeed. I want to breastfeed him* . . *. I don’t want to go back to work.* [IDI 4_ex-working mother]

From the factory perspective, the loss of trained workers was a serious problem. The RMG factory welfare officers clearly understood the struggles that mothers face but prioritized the factories’ perspective and the implications of staff resignations on business:

*Mothers struggle to breastfeed and struggle to get reliable caregivers to take care (of the kids). Almost 50% of our workers resign shortly after maternity leave. This is very bad (for business)* . . *. When an experienced operator leaves, we may replace her but production is hampered. We recruit temporary workers in anticipation (of her return)* . . *. when mothers come back (from maternity leave) and suddenly resign*. . *. her resignation hampers production*. [KII 1_welfare officer]

### Women’s perception and experience of using expressed breast milk

After exploring the breastfeeding situation among working lactating mothers, we introduced the idea of feeding expressed breast milk. Most participants (27 out of 33) had never heard of expressing breast milk. Only 3 mothers had experience hand expressing breast milk and only 2 caregivers spoke about seeing their daughters express. One caregiver said:

*(A) doctor (from their rural area) said “if you press the breast milk out, you can keep it in a ceramic bowl and leave it (to feed)* . . *. You can use for 8 hours no problem.”* [FGD 4_caregiver]

Of the 3 mothers who had expressed breast milk only 1 was able to continue the practice. The other 2 were prevented by advice from coworkers and lack of breast milk. In the words of those 2 lactating mothers:

*I tried (to express)* . . *. Nothing comes out. I don’t have enough (milk). I tried 2-3 days before joining (work)* . . *. then I stopped trying.* [IDI 4_currently working mother]

*I left breast milk for the baby for some time. Some women (at work) said “don’t leave milk like that. The baby will catch a cold.” After that I stopped (expressing).* [FGD2_currently working mother]

When we discussed the use of expressed breast milk, mothers and caregivers expressed concerns about feeding expressed breast milk. More than a third of the participants (12 out of 33) were concerned about how long the milk remained safe and were uncomfortable about the appearance of the milk. Some participants (8 out of 33) were concerned about how the expressed milk would affect the health of the child. A grandmother said:

*The milk looked reddish . . . There was (a) layer on it . . . Maybe from what she (the mother) ate. It looked bad so I threw it out. She (the mother) expressed a few more times and left (the milk) but I was not comfortable (feeding it) . . . Milk left out (for a long time) might be harmful (for the baby).* [FGD 4_ other caregiver]

## Discussion

In this study we explored knowledge about and experience of breastfeeding and structural barriers to breastfeeding at work and at home among lactating mothers working in RMG factories. Despite Bangladeshi laws mandating maternity leave, crèches and breastfeeding breaks, women working in industries such as the RMG sector have faced significant structural barriers to breastfeeding both at home and at work. As a consequence of inadequate support, often mothers had quit their jobs. Although previous studies have shown that working mothers have been impeded from adequately breastfeeding [3–6], to our knowledge this is the first published study to explore the structural barriers faced by lactating mothers working in Bangladesh’s RMG sector, the country’s largest employer of women as of 2019 [30]. The programmatic insights articulated from the study (Table 3) may help to inform future interventions and policies for breastfeeding promotion in factories in Bangladesh and other low resource setting where, increasingly, women are entering the formal workforce.

**Table 3:** Programmatic implications of the study findings

Areas of interest	Constraints/ barriers	Facilitators	Opportunities for intervention
Perceptions of breastfeeding	-Working mothers introduced formula in preparation for joining work	- All mothers prized breast milk over formula -Care givers were supportive of breastfeeding -All female RMG workers received a maternity benefit package and leave	-At the factory level policies supportive of breastfeeding should be enforced - Counseling and a supportive environment (breast pump, space, breaks) should be provided by the factories as a part of their maternity package
Perceptions of expressed breast milk	- Concerns of longevity and safety of the expressed milk -Not a social norm	- Mothers prized breast milk -Mothers and caregivers understood the disadvantages of formula	- It is important to ensure the safety of the expressed breast milk - Mothers can be trained and counseled to express and use breast milk -Caregivers should be trained about the use of expressed milk
Sources of information	-Mothers are receiving negative information about expressed breast milk from their social network and/or media	- A few doctors are promoting expressed breast milk	-There is a need for developing behavior change communication (BCC) strategies to increase the cultural acceptability of expressed breast milk at work and in the community -Key people in social network such as mothers, mothers-in-law, husbands should be targeted for counseling -BCCs should address beliefs about expressed breast milk
Work environment	- Heavy workload and limited break time - No privacy for breastfeeding - Inadequate crèche facilities -Unsupportive supervisory chain	-Factory policy for providing breastfeeding breaks -Most factories had a day care facility -Management is concerned about high employee dropout rates	-Create space in the existing crèche for breast milk expression -Create a program for breast milk expression with the time constraints in mind -Train existing crèche workers to counsel and train mothers -Motivate the factory management to invest in the program - Constantly motivate the supervisors to ensure that mothers can utilize breaks

Working mothers and other caregivers were very aware of the importance of breastfeeding for the health and survival of their infants. Many working mothers chose to quit work soon after maternity leave because of high stress and lack of support at work. When family circumstances compelled lactating mothers to keep their RMG factory jobs, they suffered from both physical pain and mental anxiety when they could not breastfeed adequately. A previous study conducted on RMG workers who left their infants and toddlers behind in rural areas showed that those working mothers suffered from anxiety and depression caused by separation from their infants [21].

Many studies have found a significant association between mothers’ return to work and duration and cessation of breastfeeding [31, 32]. Lack of workplace support has been one of the most frequently cited barriers to breastfeeding [33, 34]. In a study conducted among Chinese working mothers, researchers reported that industry related occupations were negatively associated with continued breastfeeding [35]. During qualitative exploration the researchers identified 4 work-related factors that influenced breastfeeding practices: 1) employment benefits, 2) commute time, 3) workplace environment and 4) labor intensity. With women having increasingly entered the workforce in Bangladesh [36], promoting the use of expressed breast milk among working women could offer a solution to address what has been the country’s stagnant rate of exclusive breastfeeding.

Among our participants, most lactating mothers did not know about the potential use of expressed breast milk. Of the 3 who did know, only 1 successfully practiced it. Although mothers and caregivers were supportive of feeding breast milk to infants, they were concerned about the safety of expressed breast milk, a concern supported by research. In a US study, researchers reported that pumped human milk comes in contact with 2–6 containers before feeding [37] which partly explains recent data showing widespread pathogenic contamination of pumped human milk [38, 39] and increased coughing and wheezing among infants bottle-fed human milk compared to infants fed at the breast [40]. In our population, breast pumps have not been readily available and women have not had access to refrigeration at work or home, so promoting expressed breast milk in the RMG sector will likely need the active involvement and support of the factory management and owners.

It is important to note, however, that even if breast pumps and refrigeration were provided, barriers to the use of expressed breast milk might remain. In a pilot study that supported working mothers to express breast milk in Phnom Penh, mothers were reluctant to continue the practice and cited difficulties transporting expressed milk between work and home and mistrust of the storage refrigerators at work [41]. Any program designed to promote expressed breast milk in Bangladesh, therefore, must take into account the feasibility and acceptability of the program while also considering the larger societal problem of ingrained hardships for girls and women in Bangladesh and the specific ramifications of that negative environment on breastfeeding. In view of the promotion of breast milk feeding among working mothers it is also important to explore other options such as job sharing (splitting shifts, which could allow breastfeeding), allowing part time work, improving the quality of crèches so that mothers are able to breastfeed on demand, task shifting (employing lactating women in less demanding activities) and providing longer maternity leave.

We found that many working mothers quit their jobs when faced with lack of support for breastfeeding and childcare. RMG management confirmed this finding through their expressed concerns about losing experienced workers after maternity leave. Continuity of workforce can be a crucial component of workforce competence. As RMGs have been Bangladesh’s most important industry – accounting for 83% of national exports as of 2019 [42] – the protection of competency in the RMG sector should be a national priority. Despite strong laws and policies supporting breastfeeding [43], the implementation of those laws has been seriously lacking. As a result, female workers have faced unlimited overtime, high workplace stress and physical and mental abuse from their employers [19, 20, 44]. Through inadequate support, Bangladesh has abandoned mothers in the RMG industry to the vagaries of workplace pushes for short term and shortsighted profits.

The collateral damage from prioritizing short term profits have been myriad: 1) psychological and physiological stress and ill-health for young mothers, 2) increased infant morbidity, 3) increased risk for long-term adult illnesses (e.g. diabetes, heart disease) that having been breastfed can help prevent and 4) medium- and long-term industry weakness and failing international RMG market competitiveness from losing trained, skilled workers. Further, if poverty forces mothers to stay in the workforce, we risk pushing them deeper into the cycle of poverty as they spend more money on formula and healthcare and earn less money because of absenteeism (to care for sick infants). Creating a supportive environment for working mothers in regards to breastfeeding and childcare could contribute to reaching multiple Sustainable Development Goals [45, 46] which relate to the health and wellbeing of the population. If Bangladesh as a nation is serious about human rights and girls’ and women’s rights, we need to ensure that working mothers do not need to choose between economically contributing to family income and the health and wellbeing of their children.

The strength of our study was that we conducted an in-depth exploration of experiences of breastfeeding and childcare across diverse perspectives and experiences of mothers who were currently working at, were on maternity leave from or had recently quit RMG factory jobs. There were several weaknesses to our study. We did not ask women whether they were aware of their rights regarding maternity protections and childcare or if they were satisfied with the implementation of labor laws. In future studies such questions should be explored. Ours was a study conducted in Dhaka slums among RMG factory workers and, therefore, the findings cannot be generalized to other formal and informal sectors where women work or to other urban areas. We were able to conduct interviews with factory management in a single factory, only, which may not be representative of other factories.

## Conclusions

Although lactating mothers working in the RMG sector had knowledge of the benefits of breast milk and the importance of exclusive breastfeeding for 6 months, most introduced formula as early as 2 months after delivery due to structural barriers in the workplace and at home. However, when early introduction of formula was not enough, in response to the structural barriers, mothers often quit their jobs. Between mothers’ knowledge and the existence of laws both mandating paid maternity leave and supporting breastfeeding at work, it becomes clear that it is not knowledge, but priorities placed on creating supportive environments and a more embracing view of wealth that are crucial to improving breastfeeding rates among mothers working in RMG factories.

At the time of our study, all involved parties were losing: infants were deprived of a life-saving source of health and wellbeing; mothers’ were deprived of mental peace as they were forced to choose between breastfeeding and providing desperately needed household income; RMG factories were losing skilled labor when mothers chose to quit; and the society was failing its poor mothers and their children. The RMG sector has been Bangladesh’s most important industry and its claims to lifting the poor from poverty have been integral to its image. However, without providing better support for its working mothers, those claims are hollow. The government must enforce its laws better protecting mothers in RMG factories and factory authorities must take a longer view of what wealth and prosperity mean for themselves, for the people who work for them and for the society as a whole. Only then might the RMG sector fulfill the promises to the nation.

## Data Availability

The datasets generated and/or analyzed during the current study are not publicly available based on the data access policy of icddr,b but are available from the corresponding author based upon reasonable request.

## Abbreviations

BCC: Behavior Change Communication
FGD: Focus Group Discussion
icddr,b: International Centre for Diarhhoeal Disease Research, Bangladesh
IDI: In-Depth Interview
KII: Key Informant Interview
RMG: Ready Made Garments
WHO: World Health Organization
